# Study on the Mechanism of Mechanical Properties and Wind Leakage Sealing Effect of KH570-Enhanced VAE/Cement Materials

**DOI:** 10.3390/ma18061205

**Published:** 2025-03-07

**Authors:** Qingsong Zhang, Huaqiang Sheng, Jinliang Li, Jinhu Li, Hao Zhang

**Affiliations:** 1 School of Safety Science and Engineering, Anhui University of Science and Technology, Huainan 232001, China; 2 State Key Laboratory of Mining Response and Disaster Prevention and Control in Deep Coal Mines, Anhui University of Science and Technology, Huainan 232001, China; 3 School of Safety, Taiyuan University of Technology, Taiyuan 030024, China

**Keywords:** KH570, wind leakage sealing material, modification mechanism, tensile strength, molecular dynamics simulation

## Abstract

In order to address the issue of wind leakage leading to spontaneous coal combustion in goafs during gob-side entry mining, a KH570 silane coupling agent (SCA)-modified vinyl acetate–ethylene (VAE)/cement-based flexible spraying sealing material was developed. The mechanical properties and wind leakage sealing performance of the material were evaluated using specialized testing equipment. Furthermore, molecular dynamics simulations and microstructural characterization techniques were utilized to assess and model the interface compatibility of the material. The experimental results demonstrate that KH570 significantly enhanced the material’s mechanical properties. Following modification, the material exhibited increases in the maximum tensile strength, compressive strength, and flexural strength by 53%, 38%, and 29%, respectively. KH570 not only promotes the formation of additional calcium silicate hydrate (C-S-H) gel through cement hydration, but also establishes Si-O-Si chemical bonds with cement hydration products and hydrogen bonds with the VAE emulsion. This functions as a “molecular bridge”, significantly enhancing the interface performance of the composite. The interaction between the organic and inorganic phases contributes to the formation of an interpenetrating network structure, imparting excellent compressive, flexural, and tensile deformation resistance to the material. The wind leakage of the spray-modified material was reduced by 2.7 times compared to the unmodified material, significantly improving its sealing performance under mining-induced pressure conditions. This enhancement effectively minimizes spontaneous combustion in mined-out coal areas caused by wind leakage, thereby ensuring safer mining operations.

## 1. Introduction

In 2023, China’s raw coal production reached 4.71 billion tons, accounting for 55.3% of the nation’s total energy consumption [1]. According to projections by the Chinese Academy of Engineering, by 2050, coal is expected to still constitute at least 50% of China’s primary energy production and consumption [2,3]. Therefore, coal will continue to play a significant role in the country’s energy landscape in the foreseeable future. However, coal is a non-renewable resource, and as reserves continue to deplete, the challenges related to low resource recovery and substantial waste from coal pillar mining have become increasingly prominent. An effective solution to these challenges is the adoption of gob-side entry retention mining, which yields higher recovery rates by eliminating coal pillars. Unfortunately, this method results in considerably significant wind leakage in the mined-out areas, which can trigger the spontaneous combustion of residual coal [4,5], thereby limiting the widespread adoption of this technique. Consequently, addressing issues related to wind leakage and the potential for spontaneous combustion is crucial for the broader implementation of pillar-free mining methods.

The surface spraying of sealing materials for gob-side entry retention plays a crucial role in mitigating wind leakage. Sealing materials are primarily categorized into two types: inorganic and organic materials [6]. Inorganic materials, including concrete [7,8], fly ash [9,10], and inorganic gels (e.g., silica-based gels and alkali-activated aluminosilicates) [11,12], are cost-effective and widely available. However, they exhibit poor toughness and are susceptible to breakage under external forces, thereby creating new wind leakage pathways [13]. In contrast, organic materials are characterized by controllable curing times, good adhesion, certain flexibility, and excellent sealing properties [14]. Nonetheless, organic materials are often associated with high costs, significant heat release, and flammability [15,16]. Therefore, hybridizing organic and inorganic materials provides an effective solution to these drawbacks, and numerous researchers have explored this approach [17,18,19,20,21,22,23]. Pei et al. [24] studied the impact of acrylic emulsion on the cement mortar performance and discovered that the optimal compressive and flexural strengths were obtained with an emulsion content of 16.2%. Liu et al. [25] investigated the incorporation of polyacrylamide (PAM) into concrete to enhance its flexural strength and crack resistance. The results showed that the initial fracture toughness increased by 47.5% following the addition of 7% PAM. Li et al. [26] explored the impact of styrene–butadiene rubber (SBR), styrene–acrylic emulsion (SAE), and polyacrylate emulsion (PAE) modifications on the compressive and flexural strengths of cement mortar after 7 and 28 days of curing. Their results demonstrated that the mechanical properties of the composite materials were notably enhanced after modification with the three polymers, in comparison to the control group. The polymer’s film-forming characteristics facilitate the formation of an interpenetrating network structure within the cement, thereby improving its compressive and flexural properties [27]. However, due to poor compatibility between polymers and the cement matrix, these materials often show inadequate tensile deformation resistance. Tensile deformation resistance is crucial for effective sealing in gob-side entry retention mining. Therefore, enhancing the interface compatibility between polymers and cement-based materials is essential for the development of high-performance sealing materials.

SCAs contain both organic and inorganic functional groups, allowing them to be compatible with both polymers and cement mortars, effectively resolving compatibility issues between organic and inorganic materials. Qin et al. [28] discovered that adding SCAs markedly increased the compressive strength of polyurethane/fly ash composites. Feng [29] investigated the impact of SCA modification on the properties of Kevlar fiber-cement-based materials, showing that the inclusion of SCAs significantly enhanced the adhesion between Kevlar fibers and the cement matrix, resulting in a 44.95% improvement in mechanical properties. Li et al. [30] investigated the surface modification of coal gangue (CG)–polyethylene (PE) composites with the SCA KH550, and discovered that the composite’s tensile strength increased by 74.2% when KH550 was added at 2%. These studies primarily focus on the macroscopic effects of SCAs on the mechanical properties of materials, yet there is limited systematic and targeted analysis from a microscopic perspective, particularly regarding the origin and stability of the binding forces between KH570 and organic–inorganic materials.

To address these challenges, this study uses KH570 SCA-modified VAE/cement-based materials to resolve the compatibility issues between organic and inorganic materials. A range of microscopic characterization methods, such as XRD, FTIR, and SEM, were utilized to investigate the interface performance of KH570-modified materials, uncovering the mechanism that underlies the improvement in the macroscopic mechanical properties of VAE/cement-based materials following KH570 modification. Based on these findings, molecular dynamics simulations were performed to construct KH570/VAE/C-S-H interface models. These models were used to investigate the origin and stability of the binding forces, unveiling the toughening mechanism of SCAs. Finally, spraying leakage experiments were performed on the materials before and after modification to evaluate the wind leakage sealing performance of the composite material under both dynamic and static pressure conditions.

## 2. Materials and Methods

### 2.1. Materials

1. Ordinary Portland Cement

The 42.5-grade Ordinary Portland Cement produced by Conch Group was utilized, with the main chemical composition provided in Table 1.

2. VAE Emulsion

VAE emulsion produced by Beijing Dongfang Chemical was employed with the main technical specifications presented in Table 2.

3. SCA

KH570, manufactured by Shanghai Aladdin Biochemical Technology Co., Ltd., Shanghai, China, was selected for this study. The main parameters are listed in Table 3.

4. Filler

Lightweight Calcium Carbonate: The calcium carbonate powder, manufactured by Tianjin Zhiyuan Chemical Reagent Co., Ltd. (Tianjin Shi, China), was employed. It is a white powder with a large specific surface area, non-toxic, odorless, non-irritating, and suitable for use as a food additive. Its relative density is 2.93. Lightweight calcium carbonate is used as a filler in mining thin-layer spraying materials, facilitating dispersion and enhancing adhesion.

5. Additives

Polycarboxylate superplasticizer: the polycarboxylate superplasticizer produced by Shandong Yousuo Chemical Technology Co., Ltd. (Heze, China). was selected to improve the flowability of the mixture, reduce water consumption, and enhance the material’s durability.

Defoamer: the defoamer, produced by Zhengzhou Meibo Food Technology Co., Ltd. (Zhengzhou, China), eliminates small bubbles in the thin-layer spraying material slurry, reduces porosity, and ensures the stability of the performance of the sprayed layer.

Sodium silicate: the sodium silicate produced by Gulf Group (Riyadh, Saudi Arabia) was employed as a setting accelerator, accelerating the cement hydration process and enabling rapid setting and hardening to achieve sufficient strength.

### 2.2. Material Preparation

**Figure 1:**
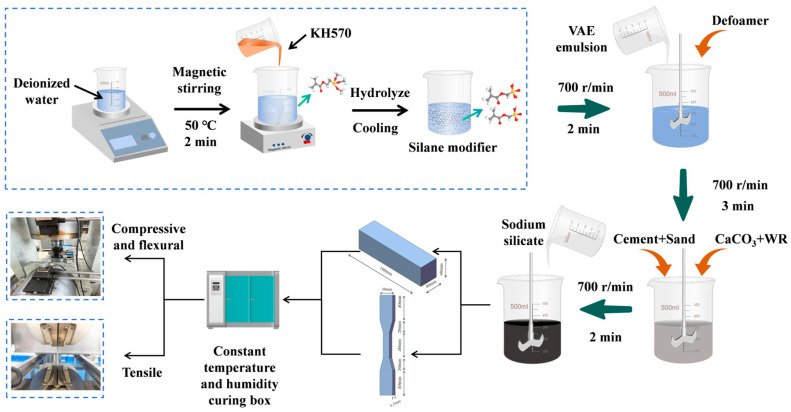
Flowchart of flexible spraying material preparation process.

The material preparation process is shown in Figure 1. Initially, a specific amount of deionized water was measured, and varying amounts of KH570 were added to the solution. The mixture was stirred at 50 °C for 2 min, then allowed to cool to room temperature. Subsequently, VAE emulsion and defoamer were added in specified proportions to the silane-modified agent, which was stirred at 700 rpm for 2 min. Cement, sand, calcium carbonate powder, and water-reducing agent (WR) were incorporated into the mixed emulsion and stirred for an additional 3 min. Finally, sodium silicate was added to the mixture and stirred for 2 min, thus completing the preparation of the composite material. The material formulation is provided in Table 4.

For compressive and flexural strength testing, rectangular specimens (40 mm × 40 mm × 160 mm) were fabricated. For tensile strength testing, dumbbell-shaped specimens were prepared. After curing in a standard cement curing box for the specified duration, the specimens were demolded and tested.

### 2.3. Testing Methods

#### 2.3.1. Mechanical Property Measurement

Based on DL/T 5126-2001 [31], rectangular specimens (40 mm × 40 mm × 160 mm) were prepared for compressive and flexural strength testing using the Rock Mechanics Testing System (RMT). Tensile test specimens were fabricated according to JC/T 2461-2018 [32], and tensile strength was measured using an electronic universal testing machine with a loading rate of 0.5 mm/min. Each set of specimens was tested three times, and the average value was used as the result.

#### 2.3.2. XRD and FTIR Analysis

Samples cured for 7 days were soaked in anhydrous ethanol for 24 h to dehydrate and stop the hydration reaction. They were then transferred to a vacuum drying oven set at 50 °C and dried until a constant weight was reached. All anhydrous ethanol was removed, and the samples were ground to 200 mesh, stored in sealed bags, and tested using XRD and FTIR.

#### 2.3.3. SEM Analysis

Samples cured for 7 days were taken from the interior of the specimen, immersed in anhydrous ethanol for 24 h, and the hydration reaction was halted, then dried in a vacuum oven set at 50 °C until a constant weight was reached, with all anhydrous ethanol removed. The surface of the materials was gold-coated, and their microstructure was observed using SEM.

#### 2.3.4. Molecular Dynamics Simulation

The mechanical properties of cement-based composites are primarily governed by the hydration products of cement, which are predominantly composed of C-S-H [33]. The commonly used Tob11 structure [34,35] was selected as the cement gel model. Interface models were constructed using Materials Studio 2020 software, followed by geometric optimization, with subsequent computations carried out using the Forcite module.

**Figure 2:**
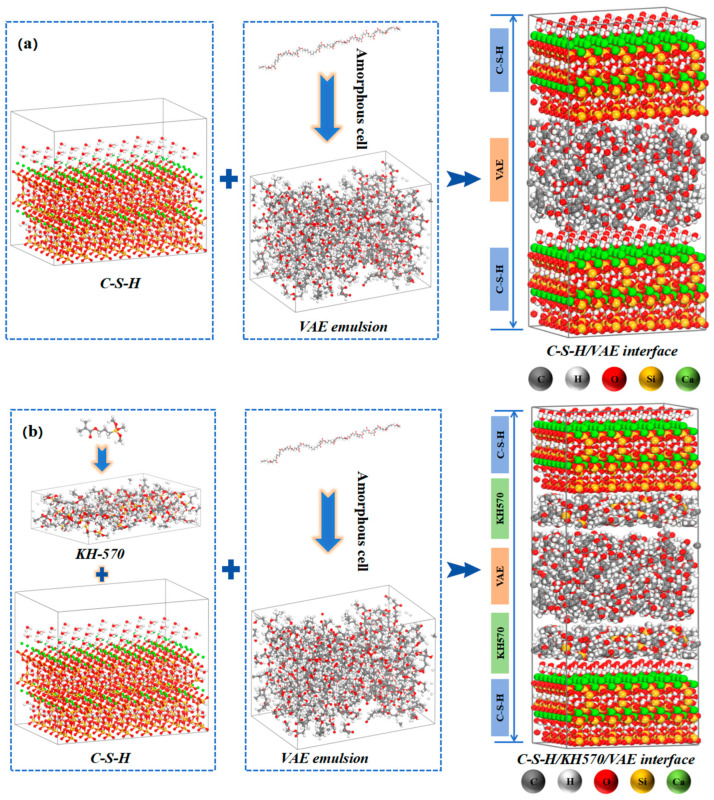
Construction of composite material interface models. (**a**) C−S−H/VAE interface; (**b**) C−S−H/KH570/VAE interface.

Figure 2a shows the C-S-H/VAE emulsion interface model, with structural dimensions of a = 45 Å, b = 30 Å, and c = 94 Å, α=β=γ=90°. Figure 2b shows the C-S-H/KH570/VAE emulsion interface model, with structural dimensions of a = 45 Å, b = 30 Å, and c = 141 Å, α=β=γ=90°. The C-S-H gel adopts the Tob11 structure, which is sliced along the [0 0 1] crystal plane and extended to form a supercell with a Ca/Si ratio of 0.67. The VAE consists of an ethylene chain made of 10 ethylene and vinyl acetate monomers (3:7 ratio), while KH570 consists of an amorphous supercell containing 36 KH570 molecules. In addition, to achieve better modeling results, the C-S-H gel in the interface model was constructed as a constrained layer, ensuring that the C-S-H and KH570-VAE layers have relatively flat surfaces along the *z*-axis. Neither VAE or KH570 were fixed to the inner surface during modeling, as they can be energy-optimized to a more stable position. Although the model only includes the main components of the material, the other components are present in much smaller quantities than in the actual material, yet still fall within a reasonable range for theoretical simulation [36].

After optimization, the model system was run under the NPT ensemble to achieve a stable state, with a balanced density and volume. Subsequently, molecular dynamics simulations were conducted under the NVT ensemble, with an initial temperature set randomly, and a simulation time and step size of 500 ps and 1 fs, respectively. The temperature controller used was Nose. All geometric optimization and MD simulations were executed using the Compass II force field.

#### 2.3.5. Wind Leakage Sealing Performance Testing

**Figure 3:**
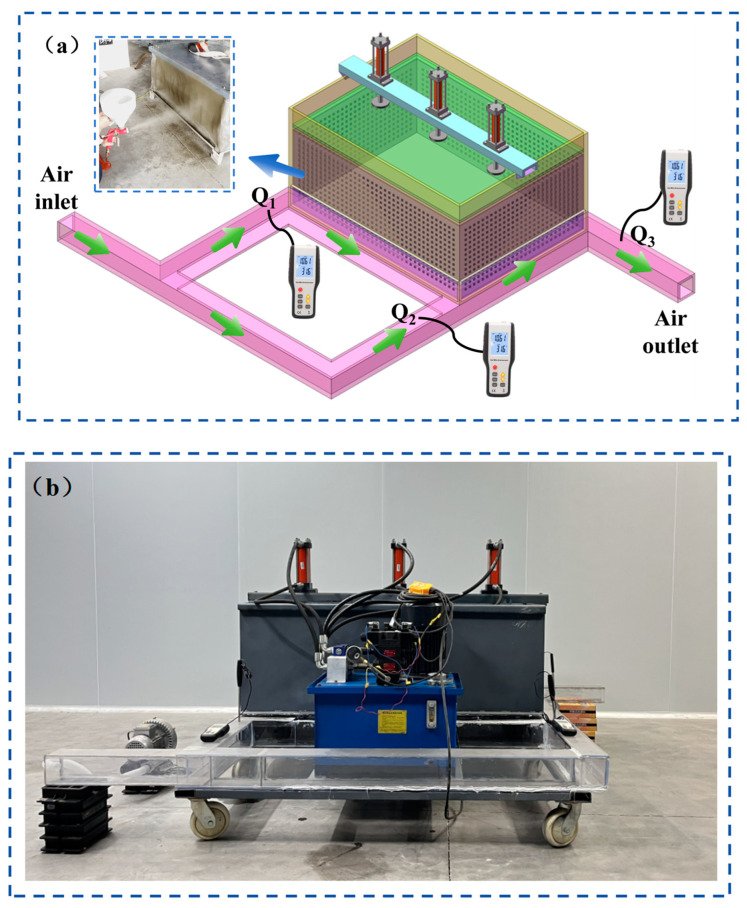
Device principle and physical diagram. (**a**) Schematic diagram of the device; (**b**) Physical prototype of the device.

A visual wind leakage sealing performance testing platform (Figure 3) was established to simulate the environment of a mined-out area in a mine. The setup includes four major systems: a filling system, airflow simulation system, wind flow measurement system, and mining pressure simulation system. The filling system consists of a frame made of iron wire mesh, with simulated fractured coal rock material inside to represent tunnel wall cracks. The airflow simulation system consists of a fan and a tunnel made of acrylic plates, forming a “two-in-one-return” Y-type ventilation system. The wind flow measurement system is made up of three HT9829 anemometers, while the mining pressure simulation system includes a hydraulic jack and pump, used to simulate the mining-induced pressure in underground mines.

## 3. Results

### 3.1. Mechanical Property Analysis

#### 3.1.1. Compressive and Flexural Strength

**Figure 4:**
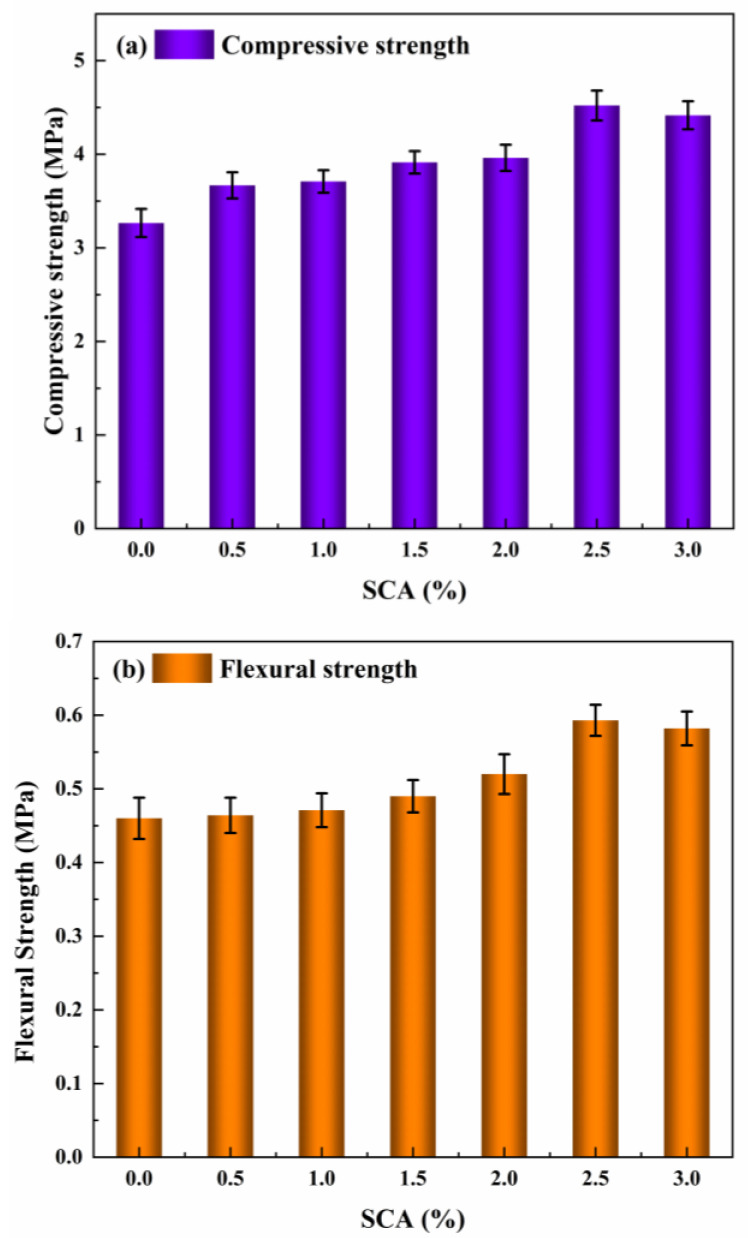
Variation of compressive and flexural strength in composite material with SCA (*w*/*w*) content. (**a**) Compressive strength; (**b**) Flexural strength.

The compressive and flexural strengths of the composite materials were measured at varying dosages of the SCA (*w*/*w*) to determine the optimal incorporation level. The experimental results are presented in Figure 4.

From Figure 4a,b, it can be observed that as the SCA content rises, both the compressive and flexural strengths of the composite material initially increase, followed by a subsequent decrease. When the SCA content reached 2.5%, the compressive strength peaked at 4.521 MPa, and the flexural strength reached 0.593 MPa, corresponding to increases of 38% and 29%, respectively. This enhancement is primarily attributed to the SCA’s ability to reduce or eliminate the delamination and aggregation of organic and inorganic materials, thereby improving the interface compatibility and enhancing the composite material’s mechanical properties [36,37,38]. However, when the SCA content increased to 3%, a slight decline in mechanical properties was observed, in agreement with the results of similar studies [28]. This decrease could be due to the increased amount of water generated during the hydrolysis process as the SCA content increases, resulting in a higher water-to-cement ratio. Once the optimal water-to-cement ratio is exceeded, the amount of cement particles per unit volume of slurry decreases relative to the spacing between particles. The colloids produced during hydration are insufficient to fill the gaps between particles. Additionally, excessive water evaporation causes the formation of additional pores within the material, which in turn leads to a reduction in strength at the macroscopic level.

#### 3.1.2. Tensile Strength

**Figure 5:**
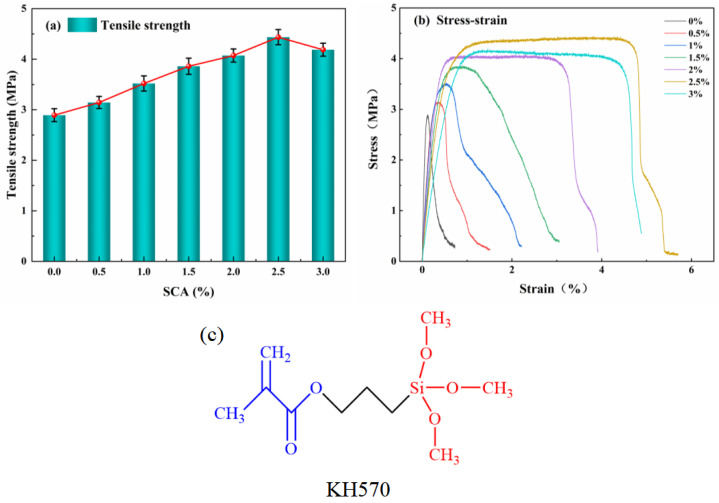
Variation of tensile strength in composite material with SCA (*w*/*w*) content. (**a**) Tensile strength; (**b**) Stress−strain; (**c**) The chemical structure of KH570.

Tensile strength plays a critical role in determining the composite material’s ability to withstand mining-induced pressure. However, the overall tensile strength of the material is relatively low, primarily due to the poor compatibility at the interface between the organic and inorganic phases. Figure 5c shows the chemical structure of KH570, where the red region represents the hydrophilic functional groups that can form bonds with the inorganic cement matrix, while the blue region represents the hydrophobic functional groups, which interact with organic components such as VAE emulsion. These interactions are crucial for the improvement of the material’s interface performance, as the amphiphilic structure allows for better compatibility between the organic and inorganic phases. As a result, by varying the dosage of the SCA and assessing its impact on tensile strength, the optimal concentration was identified. The relationship between the SCA dosage and the resulting tensile strength of the composite material is illustrated in Figure 5.

As depicted in Figure 5a, the tensile strength of the composite material initially increases with the rising content of SCA, but this trend reverses after a certain point. Specifically, at a SCA concentration of 2.5%, the tensile strength reaches its maximum value of 4.436 MPa, reflecting a 53% improvement over the unmodified material. This pattern suggests that there is an optimal dosage beyond which further increases in the SCA content may lead to a decrease in performance. Similarly, Figure 5b demonstrates a clear influence of the SCA content on the stress–strain characteristics of the composite. When no SCA was incorporated, the material exhibited a strain at break of only 0.73%. However, as the concentration of SCA increased, the strain at break progressively improved, reaching a peak of 5.7% at 2.5%, which represents a sevenfold increase compared to the unmodified version. This significant enhancement in the deformation capacity is attributed to the modification of the composite structure, in which the SCA induces a transition from tensile softening to tensile hardening behavior [39]. Despite these improvements, when the SCA concentration reached 3%, both the tensile strength and strain at break showed a slight decline. This decrease can be explained by the increased water produced during the hydrolysis process of the SCA, which raises the overall water-to-cement ratio beyond the optimal threshold. As a result, the excess water leads to a slight reduction in the internal hydration products, diminishing the tensile performance at the macroscopic scale. This observation underscores the importance of maintaining a balanced water-to-cement ratio to maximize the mechanical properties of the composite material.

### 3.2. FTIR Analysis

**Figure 6:**
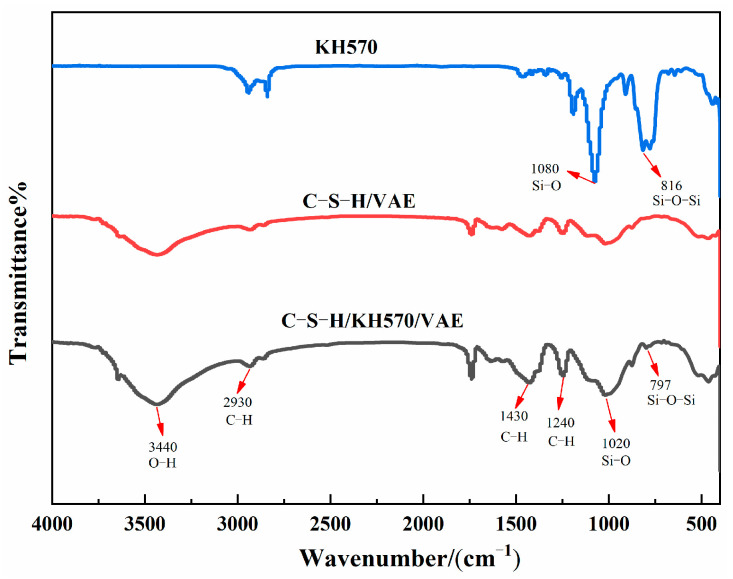
Infrared spectrum of the composite material before and after modification.

The SCA enhances the compatibility between the organic and inorganic components. FTIR analysis can be employed to examine the interaction between the organic and inorganic structures and the SCA, revealing changes in chemical bonds during the reaction and aiding in the understanding of the underlying reaction mechanism. The experimental results are presented in Figure 6.

As shown in Figure 6, the peak observed at 3440 cm^−1^ corresponds to the stretching vibration of the −OH group, while the peaks at 2930 cm^−1^ and 1240 cm^−1^ are associated with the stretching vibration of −CH_3_ in the VAE emulsion. Additionally, the peak at 1430 cm^−1^ is attributed to the bending vibration of the −CH_2_ group in the VAE emulsion. These changes arise from chemical reactions occurring on the surface of the cement after the incorporation of VAE. The peaks at 1080 cm^−1^ and 1020 cm^−1^ correspond to the stretching vibration of the Si−O bond [36]. In the cement/VAE/KH570 composite, a new peak appeared at 797 cm^−1^, which is linked to the stretching vibration of the Si−O−Si bond at 816 cm^−1^. This observation suggests that the SCA actively participates in the cement hydration process, where hydrolysis of the SCA generates silanol groups, which then condense with the silanol groups on the cement surface, forming Si−O−Si bonds. This confirms that the SCA acts as a molecular bridge, which is a key factor in the enhanced tensile and compressive strength of the modified material. The detailed mechanism of action of the SCA is schematically illustrated in Section 3.6.

### 3.3. XRD Analysis

**Figure 7:**
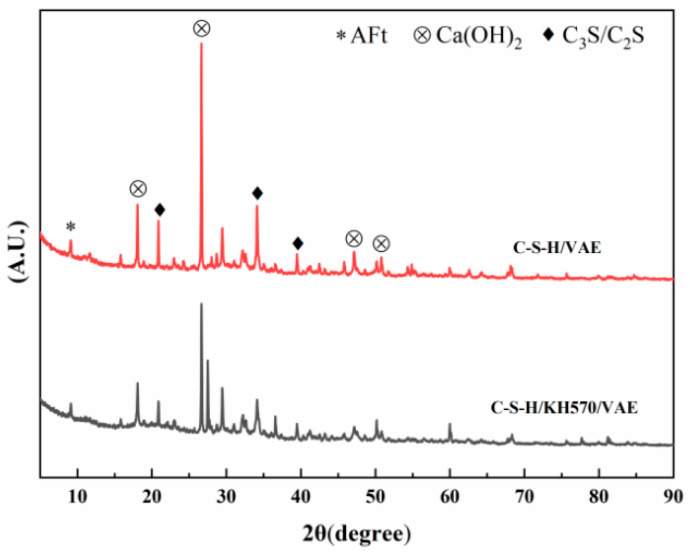
XRD patterns of the composite material before and after modification.

To analyze the impact of the SCA on the inorganic structure of the composite material, XRD testing was used to determine whether new phases appeared after modification, and it was also employed to measure the microscopic stress within the material. The experimental results are shown in Figure 7.

As shown in Figure 7, when no SCA was added, the main products after the reaction of the VAE/cement composite were ettringite (AFt), Ca(OH)_2_, and unhydrated tricalcium silicate (C_3_S) and dicalcium silicate (C_2_S). After modification with KH570, no new diffraction peaks appeared in the XRD, and the positions and shapes of the absorption peaks were essentially consistent with those before modification. This indicates that the addition of the SCA has a minimal effect on the types of hydration products. Additionally, from Figure 7, it can be observed that although the SCA did not affect the type of products, there was a significant change in the peak intensities before and after modification. The characteristic peaks of Ca(OH)_2_ and unhydrated C_3_S and C_2_S showed a noticeable decrease in intensity. This phenomenon may be attributed to the reaction of KH570 with cement to form Si-O-Si chemical bonds, which effectively promotes the hydration of unhydrated C_3_S and C_2_S to form C-S-H gel, while also reducing the amount of Ca(OH)_2_, which has a significant impact on cement hydration, ultimately enhancing the mechanical properties of the composite material [40].

### 3.4. SEM Analysis

**Figure 8:**
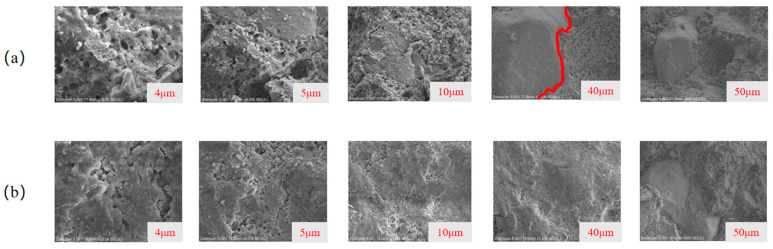
Microstructure of the material at different scales: (**a**) Cement-VAE emulsion system; (**b**) Cement-VAE emulsion-KH570 system.

SEM can be used to observe the surface roughness, cracks, pores, and other microstructural features of the material. By comparing the surface crack patterns before and after modification, the enhancement of interface compatibility between the organic and inorganic phases due to the SCA can be observed at different scales. The experimental results are shown in Figure 8.

From Figure 8a, it can be observed that at a 40 μm scale, a distinct boundary appears in the cement-VAE emulsion system, indicating poor interface compatibility between the cement and emulsion, with some cement not reacting with the emulsion. From Figure 8b, it can be seen that after the addition of the SCA, no distinct boundary is observed. The material appears dense and smooth, suggesting that the SCA participated in the cement hydration reaction, acting as a molecular bridge and significantly enhancing the interface compatibility. Additionally, from Figure 8b, it can be observed that at scales of 4 μm, 5 μm, and 10 μm, after the addition of the SCA, the internal porosity of the composite material was significantly reduced, and the surface became denser. At the 50 μm scale, the material became more uniform and smooth, with a significant reduction in porosity. This indicates that the addition of the SCA strengthened the bonding between the VAE emulsion and cement hydration products, which macroscopically resulted in improved mechanical properties.

### 3.5. Interface Structure Analysis

The mechanical properties of composite materials are determined by both their structural and dynamic characteristics [41]. The static analysis of the model involves the relative concentration distribution (RCD) and the radial distribution function (RDF). The RCD provides insights into the atomic density distribution along a specified direction, thereby revealing the local structure between the modifier and the cement material. The RDF, on the other hand, is used to further investigate the interactions and microscopic arrangement of the particles. The dynamic analysis of the model focuses on the mean square displacement (MSD), which quantifies the dynamic behavior of the composite material following the addition of the SCA.

#### 3.5.1. Static Structure Analysis

##### RCD

**Figure 9:**
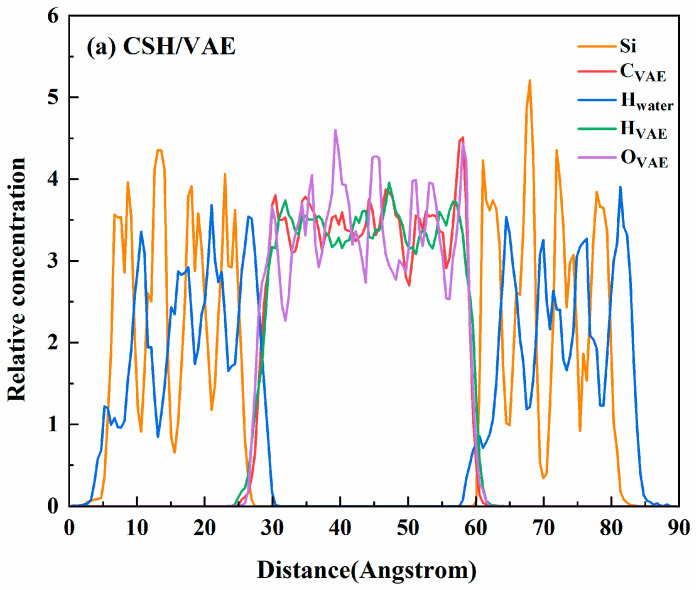

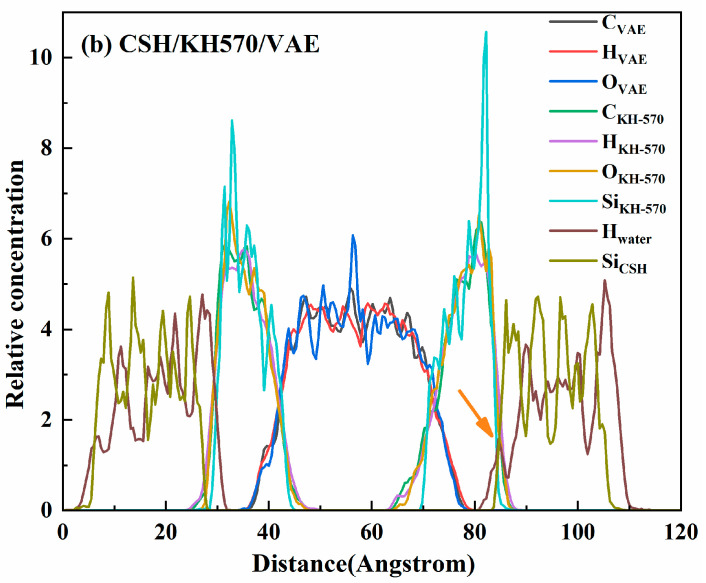
Relative concentration distribution of atoms. (**a**) RCD of C−S−H/VAE; (**b**) RCD of C−S−H/KH570/VAE.

The interface model before and after KH570 modification is a layered structure constructed along the *Z*-direction (0 0 1). Therefore, the intensity distribution of different atoms along the direction perpendicular to the interlayer (i.e., the *Z*-direction) was statistically analyzed. The results are shown in Figure 9.

From Figure 9, it can be observed that multiple strong peaks are present for Si and H_2_O molecules within the C-S-H structure, and some of the H_2_O peaks lie between two peaks of Si atoms, indicating a strong affinity of the C-S-H layered structure for water molecules. From Figure 9a, it can be observed that in the polymer emulsion/cement-based system, the atomic concentration distribution is nearly symmetric, indicating that the chemical reaction between the polymer and the cement material is minimal in this system. From Figure 9b, it can be seen that the addition of the SCA disrupts this symmetry, intensifying the chemical reaction within the system. An overlap of Si_KH570_ and Si_C-S-H_ occurs at 85 Å, suggesting the possible formation of Si-O-Si chemical bonds within the composite material system.

##### RDF

**Figure 10:**
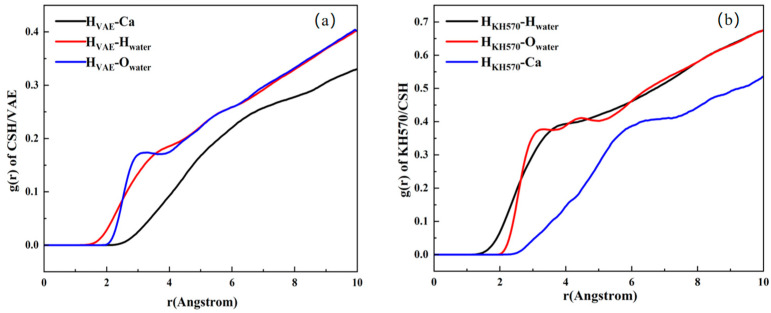

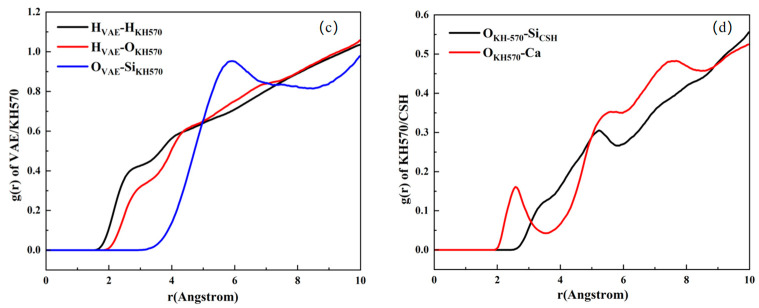
RDF distribution of different systems. (**a**) RDF of the major atoms in C−S−H/VAE; (**b**) RDF of KH570 with C−S−H in C−S−H/KH570/VAE; (**c**) RDF of VAE with KH570 in C−S−H/KH570/VAE; (**d**) RDF of the major atoms in KH570/C−S−H.

To gain deeper insights into the interface structure between C-S-H, VAE, and KH570, RDF calculations were conducted using simulation software, correlating microscopic features with macroscopic properties. The location of the first characteristic peak in the RDF curve indicates the bond length of the chemical bonds [42]. The results are presented in Figure 10.

Figure 10a presents the RDF curve for the C-S-H/VAE system before modification. The RDF curve of H_VAE_-O_water_ exhibits a peak at 3 Å, which indicates the formation of hydrogen bonds between the water molecules in VAE and the C-S-H structure. Figure 10b–d display the RDF curves for the modified systems. In Figure 10b, both the H_KH570_-O_water_ and H_KH570_-Ca curves show distinct peaks, suggesting the formation of hydrogen bonds and ionic bonds between the SCA and C-S-H. In Figure 10c, a prominent peak at 6 Å in the RDF curve of O_VAE_-Si_KH570_ points to the establishment of hydrogen bonds between VAE and the SCA. Figure 10d reveals a notable peak at 5 Å, corresponding to the formation of Si-O-Si chemical bonds between the oxygen atoms in KH570 and the silicon atoms in C-S-H. Additionally, a significant peak at 2.2 Å is observed, attributed to the ionic bond between the oxygen atoms in KH570 and the calcium ions in C-S-H. Overall, Figure 10 demonstrates that KH570 forms not only chemical and hydrogen bonds with the cement hydration products, but also hydrogen bonds with VAE. In the material system, KH570 acts as a molecular bridge, improving the compatibility between the organic and inorganic phases and linking the organic and inorganic components.

#### 3.5.2. Dynamic Structure Analysis

##### MSD

**Figure 11:**
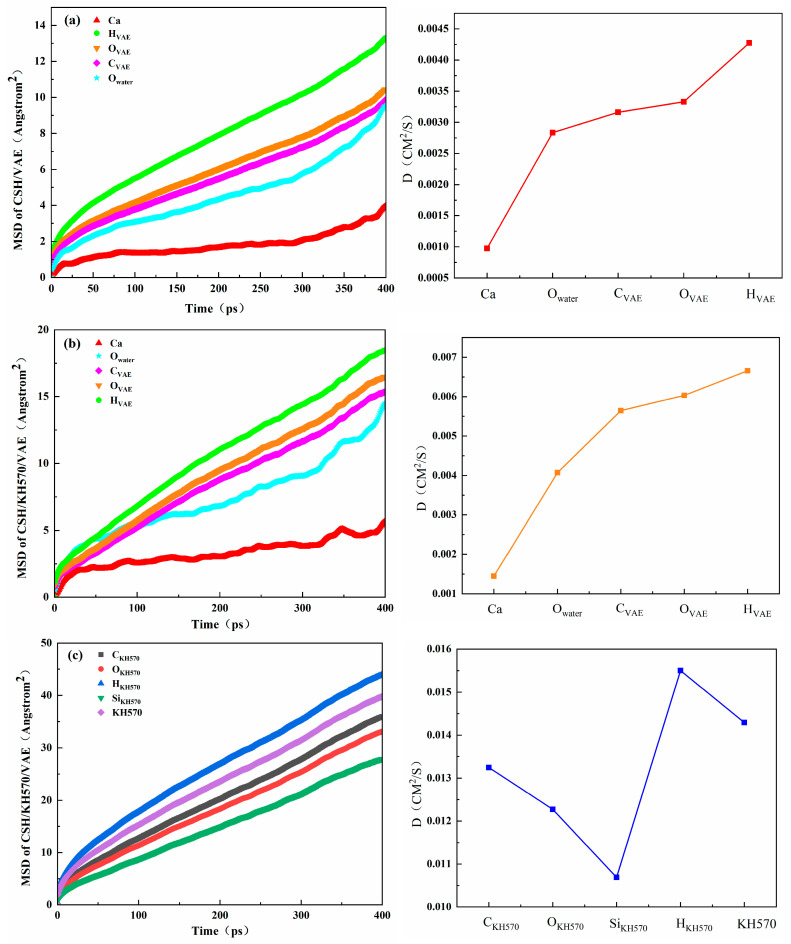
(**a**) MSD variation of main atoms in the C-S-H/VAE system; (**b**) MSD variation of main atoms in the C-S-H/KH570/VAE system; (**c**) MSD variation of main atoms in KH570.

In addition to examining the static structural characterization of the model system, the dynamic properties are also crucial for evaluating the mechanical performance of the composite material. By analyzing the system’s dynamic behavior using MSD, the stability of VAE and KH570 in the cement interface connection is more intuitively assessed [43]. In this experiment, the dosage of KH570 was 2.5 wt%, and the experimental results are presented in Figure 11.

Due to the stable framework structure of C-S-H, the diffusion coefficients of Ca atoms within C-S-H are relatively low. In Figure 11b, the diffusion coefficients of the primary atoms are higher compared to those in Figure 11a, as the incorporation of KH570 enhances the free movement of atoms at the interface, thus increasing atomic interactions. This also accounts for the formation of hydrogen bonds (H_VAE_-O_KH570_) observed in Figure 10c, which further improves the interface stability. Figure 11c provides an analysis of the atomic diffusion coefficients of KH570. The results indicate that the diffusion coefficients of silicon and oxygen atoms in KH570 are the lowest, which supports the presence of Si-O-Si chemical bonds between KH570 and C-S-H, effectively restricting the movement of Si and O atoms in KH570, while the KH570 atoms remain relatively mobile.

### 3.6. Toughening Mechanism of SCA Modification

**Figure 12:**
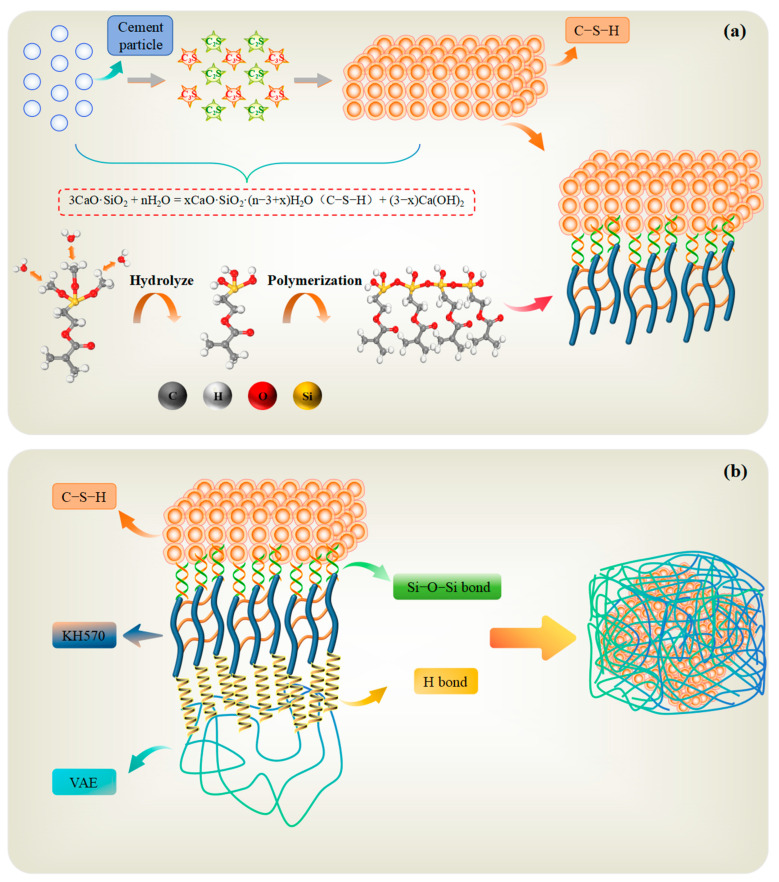
Organic–Inorganic hybrid toughening mechanism. (**a**) Mechanism of interaction between C−S−H and KH570; (**b**) Mechanism of KH570 enhancing interfacial compatibility.

Both macro-performance tests and microstructural analysis demonstrate that the incorporation of the SCA significantly enhances the overall mechanical properties of the composite material. When combined with the results from molecular dynamics simulations, the toughening mechanism of the SCA in improving the mechanical properties of the VAE/cement material is depicted in Figure 12.

Upon adding the SCA to the VAE/cement material, the cement reacts within the slurry to form C_3_S and C_2_S, which then rapidly hydrate to produce C-S-H gel and a small amount of Ca(OH)_2_. Simultaneously, the alkoxy groups in KH570 hydrolyze to generate silanol groups, which self-polymerize, transforming the SCA from its monomeric form into a network structure. The hydrophilic silanol groups undergo dehydration condensation with the silanol groups on the surface of the cement hydration product C-S-H, forming Si-O-Si chemical bonds. In this process, KH570 effectively promotes the hydration of unhydrated C_3_S and C_2_S to generate C-S-H gel, thereby enhancing the cement strength. At the same time, it reduces the amount of Ca(OH)_2_, which plays a significant role in cement hydration, further improving the compressive and flexural strength of the composite material.

The SCA modifies not only the inorganic components of the material, but also the organic components. As illustrated in Figure 6 and Figure 10, the organic functional groups in KH570 form hydrogen bonds with the VAE emulsion, facilitating the inorganicization of the VAE emulsion surface and shifting its properties from lipophilic to hydrophilic. Moreover, the organic functional groups in KH570 interconnect with the organic functional groups in the VAE emulsion, enhancing the bond between KH570 and the VAE emulsion, ultimately leading to the formation of an SCA film on the VAE surface.

Thus, the incorporation of KH570 into the VAE/cement material performs a dual modification function. The SCA acts as a “molecular bridge”, linking the VAE emulsion surface and the cement C-S-H matrix, forming a network structure that significantly improves the physical adhesion between the VAE emulsion and the cement matrix. This network structure, created by the “molecular bridge”, also exhibits a strong deformation capacity, endowing the material with exceptional toughness and deformability.

Overall, the inclusion of KH570 effectively promotes cement hydration to produce C-S-H gel, which forms Si-O-Si chemical bonds with the cement. Additionally, KH570 forms hydrogen bonds with VAE, greatly enhancing the interface properties of the composite material. This compensates for the limitation where the VAE emulsion and cement phases are originally connected only through non-bonding interactions. The internal structure of the material becomes more uniform, with significantly reduced porosity and tightly interconnected components, leading to a marked improvement in the compressive, flexural, and tensile strength of the composite material. This enhanced material can effectively meet the requirements for wind leakage sealing in gob-side entry retention mining under mining-induced pressure conditions.

### 3.7. Wind Leakage Sealing Performance Analysis

**Figure 13:**
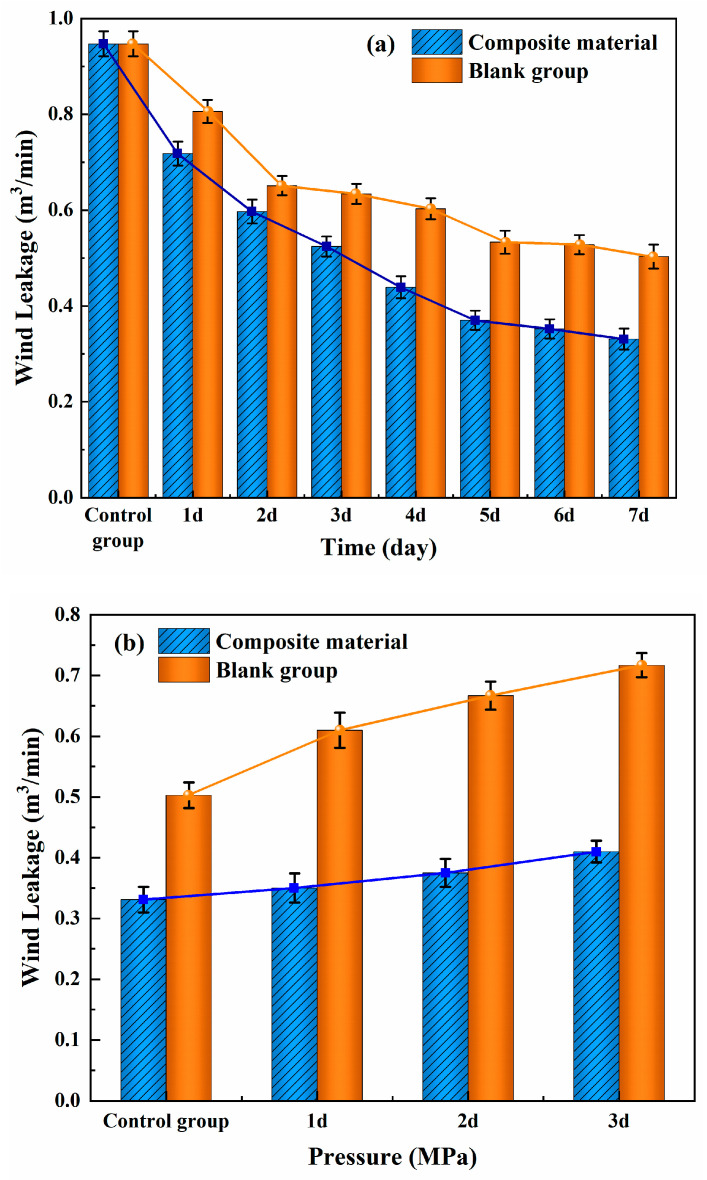
Wind leakage sealing performance of flexible spraying material. (**a**) Variation of wind leakage rate with time; (**b**) Variation of wind leakage rate with pressure.

Due to the dynamic influence of mining pressure, the gob-side entry retention often experiences repeated pressure impacts, leading to fractures and the formation of wind leakage channels. Therefore, the dynamic toughness of the material is crucial for effective wind leakage sealing. Wind leakage experiments were conducted using a self-built experimental setup to measure the variation in the wind leakage rate with the time and mining pressure to evaluate the sealing performance of the composite material under conditions of mining-induced pressure. The blank sample refers to the unmodified material, the composite material refers to the modified material, and the control sample refers to the leakage rate of the material without spraying. The experimental results are shown in Figure 13.

From Figure 13a, it can be observed that the wind leakage rate for both materials after spraying decreases with time. The leakage rate of the composite material on day 7 was 0.331 m^3^/min, while the blank material’s leakage rate on day 7 was 0.503 m^3^/min. The leakage rate of the composite material decreased by 51.8% after modification, indicating that the modified material has a better sealing performance than the unmodified material. This is primarily due to KH570’s effective promotion of the hydration reaction, particularly the formation of C-S-H gel, while also improving the interface compatibility between organic and inorganic materials. Both factors contribute to the reduction of internal porosity and a more tightly connected internal structure, significantly reducing the wind leakage during the curing process.

In the current study, the pressure range of 0–0.5 MPa was chosen for evaluating the wind leakage sealing performance of the composite material under simulated mining conditions. This pressure range reflects the typical pressures that can be encountered in goafs and mined-out areas during gob-side entry mining, where mining-induced pressures fluctuate depending on various factors such as the depth of mining, the method of extraction, and surrounding geological conditions. The simulated pressure range used in our experiments was selected to represent moderate mining-induced pressure conditions that are commonly observed in many mining environments.

It is important to note that actual pressures in underground mining can often exceed 0.5 MPa, especially in deep mines or in regions under high geostatic pressure. For instance, in deep coal mines, mining-induced pressures can reach several MPa, depending on the specific geological formations and depth of the mine. However, the pressure range used in our study is still considered relevant for assessing the sealing performance of the material under conditions typically found in medium-depth mining operations.

The choice of 0–0.5 MPa in our study was to ensure that the material’s performance is evaluated under conditions that are representative of the majority of mining operations. While higher pressures may be encountered in deeper mines, the sealing performance of materials at lower pressures provides important insight into their behavior under more typical operational conditions. Future studies could explore the material’s performance under higher pressures to further evaluate its behavior in deep mine environments, where higher mining-induced pressures are more common.

From Figure 13b, it can be seen that the wind leakage rate of both materials increases with pressure, but the increase in the leakage rate is minimal for the modified material. This is primarily because the modification allows the formation of a network-like “molecular bridge” between the cement and the VAE emulsion, imparting a good deformation ability to the material. When subjected to mining-induced pressure, the material can deform in response to pressure and cracks, thereby maintaining its integrity and preventing the formation of extensive wind leakage channels that could trigger coal spontaneous combustion.

## 4. Conclusions

A flexible spraying sealing material was developed and its performance was analyzed using macro-performance testing, microstructural analysis, and molecular dynamics simulations at multiple scales. The mechanism by which the SCA of KH570 influences the interface properties of VAE emulsion–cement composites is explained. The main conclusions drawn are as follows.

The addition of the KH570 significantly enhanced the mechanical properties of the composite material. At an incorporation level of 2.5%, the composite material achieved an optimal mechanical performance, with the maximum tensile strength increased by 53%, the strain at break increased nearly sevenfold, compressive strength increased by 38%, and flexural strength increased by 22%.

KH-570 effectively accelerates the hydration of cement in the VAE/cement composite, facilitating the formation of C-S-H gel. During hydrolysis, KH570 generates silanol groups, which then undergo dehydration condensation with the silanol groups on the C-S-H surface, resulting in the formation of Si-O-Si chemical bonds. Concurrently, the organic functional groups in KH570 establish hydrogen bonds with the VAE emulsion. The KH570 serves as a “molecular bridge”, linking the surface of the VAE emulsion to the cement C-S-H matrix. This interaction creates a network structure that considerably improves the interface properties of the composite material, providing it with outstanding compressive, flexural, and deformation resistance.

The modified flexible spraying material effectively deforms in response to mining-induced pressure, exhibiting good crack resistance. After modification, the wind leakage increment decreased by 271% compared to the unmodified material, significantly reducing wind leakage in mined-out areas and effectively lowering the probability of coal spontaneous combustion caused by secondary wind leakage.

## 5. Discussions

### 5.1. Limitations and Future Works

While this study focuses on KH570, the interfacial coupling strategy (i.e., chemical bridging and dynamic hydrogen bonding) is potentially transferable to other SCAs with tailored functional groups. Future work will systematically investigate the effects of varying SCAs (e.g., amino– or epoxy–silanes), environmental conditions (e.g., humidity and temperature), and matrix materials (e.g., geopolymers and epoxy resins) to validate the broader applicability.

### 5.2. Environmental and Ecological Considerations

(1) Environmental impact of KH570

KH570, a silane coupling agent, is widely used in various industrial fields such as adhesives, coatings, and sealants. According to the existing literature, KH570 is considered a low-toxicity chemical with minimal environmental risk, particularly when used in controlled amounts. However, the potential environmental hazards of silane coupling agents (including KH570) are primarily associated with their production, transport, and disposal phases. During large-scale production or application, KH570 may potentially enter the environment via wastewater or emissions. Therefore, the strict control of its production and handling processes is necessary to prevent chemical leakage. We suggest that more environmental risk assessments be conducted in the future to evaluate the long-term environmental impact of KH570 in practical applications.

(2) Biodegradability and disposal of KH570-modified composites

The environmental sustainability of KH570-modified VAE/cement composites is not only determined by the use of KH570, but also by the nature of the cement-based composite materials. Traditional cement materials degrade slowly in natural environments. We anticipate that KH570-enhanced cement composites may also face challenges in terms of complete biodegradability over time. It is crucial to consider the degradation performance of these composites after disposal, especially in terms of any hazardous chemicals potentially released during disposal. Hence, further life-cycle assessments and systematic environmental studies should be conducted to ensure the ecological sustainability of these composites.

(3) Recycling potential of modified composites

When considering the environmental impact of KH570-modified composites, we must also explore their recycling potential. Compared with traditional cement materials, KH570-enhanced composites offer a longer service life, thereby reducing resource consumption and the need for frequent material replacements. In mining applications, an enhanced durability and sealing performance will reduce the need for maintenance and minimize the environmental burden. Future research on recycling technologies and investigating the potential for reusing these composites after their service life will significantly contribute to reducing waste generation and promoting more efficient resource utilization.

(4) Environmentally friendly manufacturing processes

While KH570-enhanced VAE/cement composites offer various advantages, we also acknowledge the importance of adopting environmentally friendly production processes during their large-scale manufacture. For example, energy-efficient and low-pollution green manufacturing techniques should be considered to reduce the environmental impact during production. Furthermore, using sustainable raw materials, such as substituting part of the traditional cement with industrial by-products like fly ash, could greatly reduce carbon emissions and enhance the environmental friendliness of these materials.

(5) Environmental benefits in mining applications

From the perspective of mining, the environmental benefit of KH570-modified composite materials lies in their ability to effectively reduce wind leakage and the risk of coal spontaneous combustion, leading to safer mining operations and minimizing environmental pollution caused by mining accidents. Although the primary focus of our study has been the material’s performance, its application in mining offers positive environmental benefits as well.

In conclusion, while KH570-modified VAE/cement composites provide an excellent performance, their environmental impact should also be carefully considered. We suggest conducting a more comprehensive life-cycle analysis (LCA) to evaluate their environmental impact during production, use, and disposal. Additionally, future research could focus on developing green manufacturing processes, improving biodegradability, and exploring recycling potential to achieve more sustainable material development and application.

## Figures and Tables

**Table 1:** Chemical composition of cement.

CaO	SiO_2_	Al_2_O_3_	Fe_2_O_3_	MgO	SO_3_	Other
58.34%	25.16%	6.12%	4.23%	2.87%	2.12%	1.16%

**Table 2:** Technical specifications of VAE emulsion.

pH	Solid Content %	Viscosity (mPa·s)	Grain Diameter μm	Ethylene Content %
4.0–6.0	54.5	500–1000	2	16 ± 2

**Table 3:** Main parameters of KH570.

Density (g/cm^3^)	Refractive Index	Flash Point °C	Boiling Point °C	Purity %
1.045	1.429	90	250	97

**Table 4:** Composite material formulation.

NO.	W/C	C/S	Sodium Silicate/%	Calcium Carbonate/%	Defoamer/%	Water Reducer/%	VAE/%	KH570/%
1	0.4	2	2	3	0.5	0.1	20	0
2	0.4	2	2	3	0.5	0.1	20	0.5
3	0.4	2	2	3	0.5	0.1	20	1
4	0.4	2	2	3	0.5	0.1	20	1.5
5	0.4	2	2	3	0.5	0.1	20	2
6	0.4	2	2	3	0.5	0.1	20	2.5
7	0.4	2	2	3	0.5	0.1	20	3

Note: The water-to-cement ratio (W/C) refers to the mass ratio of deionized water to cement, and the cement-to-sand ratio (C/S) refers to the mass ratio of cement to sand. The contents of water glass, calcium carbonate, defoamer, VAE emulsion, superplasticizer, and SCA are expressed as percentages of the mass of cement.

## Data Availability

The original contributions presented in this study are included in the article. Further inquiries can be directed to the corresponding authors.
